# Small intestine duplication cyst with recurrent hematochezia: a case report and literature review

**DOI:** 10.1186/s12876-021-01627-6

**Published:** 2021-06-01

**Authors:** Zhicheng Zhang, Xiaowei Huang, Qian Chen, Demin Li, Qi Zhou, Jinjin Huang, Yongdong Feng, Junbo Hu, Hua Qin

**Affiliations:** 1 Department of Gastroenterology, Tongji Hospital, Tongji Medical College, Huazhong University of Science and Technology, Wuhan, 430030 China; 2 Department of Hematology, Tongji Hospital, Tongji Medical College, Huazhong University of Science and Technology, Wuhan, China; 3 Department of Gastrointestinal Surgery Center, Tongji Hospital, Tongji Medical College, Huazhong University of Science and Technology, Wuhan, China

**Keywords:** Small intestine duplication cyst, Hematochezia, Case report

## Abstract

**Background:**

Small intestine duplication cysts (SIDCs) are rare congenital anatomical abnormalities of the digestive tract and a rare cause of hematochezia.

**Case presentation:**

We describe an adult female presented with recurrent hematochezia. The routine gastric endoscope and colonic endoscope showed no positive findings. Abdominal CT scan indicated intussusception due to the "doughnut" sign, but the patient had no typical symptoms. Two subsequent capsule endoscopes revealed a protruding lesion with bleeding in the distal ileum. Surgical resection was performed and revealed a case of SIDC measuring 6 * 2 cm located inside the ileum cavity. The patient remained symptom-free throughout a 7-year follow-up period.

**Conclusion:**

SIDCs located inside the enteric cavity can easily be misdiagnosed as intussusception by routine radiologic examinations.

## Background

Enteric duplication cysts (EDCs) are rare congenital anatomical abnormalities of the digestive tract with an incidence of one in 4500 [1]. EDCs are found throughout the digestive tract, with nearly half occurring in the small intestine [2]. Even though rare in adults, they are most commonly found in infants. EDCs are typically located at the mesenteric boundary of the digestive tract [1].

Hematochezia is a difficult matter in clinics and may even be life-threatening. Causes for hematochezia include peptic ulcer, Meckel’s diverticulum, intussusception, and EDC. Sometimes, differential diagnosis is difficult. Small intestine duplication cysts (SIDCs) were mostly located outside the digestive tract [3]. Notably, we observed a case of SIDC inside the distal ileum and it was an unusual source of hematochezia.

## Case presentation

**Fig. 1:**
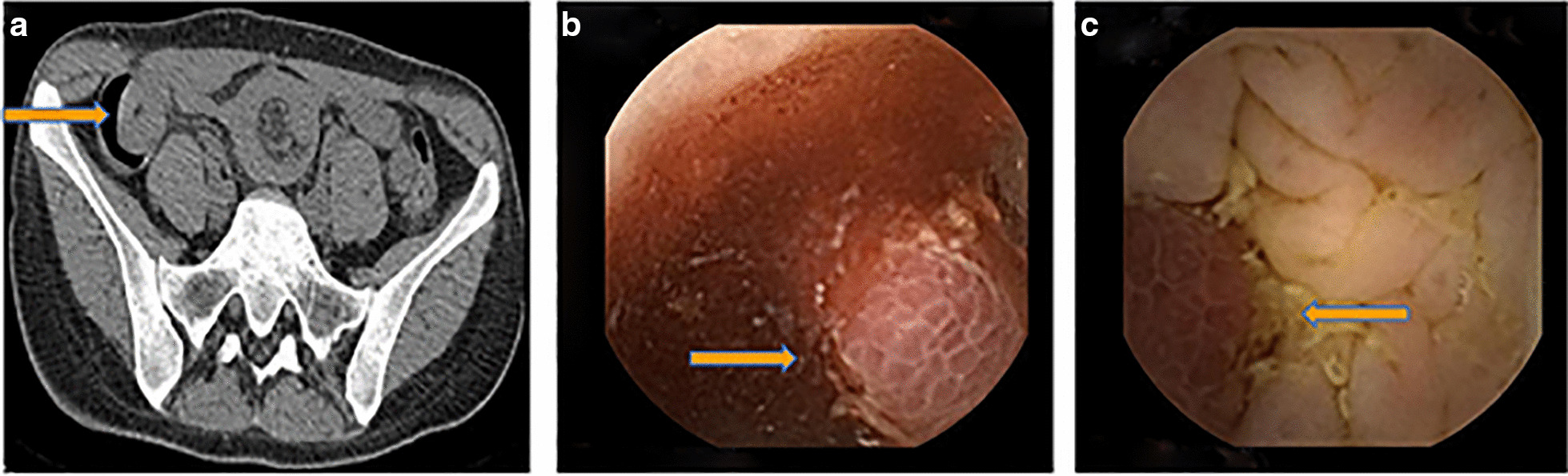
CT and capsule endoscope images of the lesion. Abdominal CT showed an indistinct ileocecal structure with a “doughnut” sign (yellow arrow) (**a**). Two subsequent capsule endoscopes revealed a protruding lesion with bleeding in the distal ileum(yellow arrows) (**b**, **c**)

A 31-year-old woman was admitted with intermittent hematochezia for a month and a recurrence three days ago. She has no special past medical history. Physical examination revealed no abnormalities with stable vital signs. Hemoglobin levels varied between 50 and 90 g/L (normal range: 110–150 g/L), and fecal occult blood testing was positive. Routine coagulation indicators and tumor markers, including carcinoembryonic antigen (CEA) and carbohydrate antigen 19–9 (CA19-9), were within normal ranges. The rapid and massive hematochezia resulted in shock after admission. After initial resuscitation, the patient underwent gastric endoscope and colonoscopy examinations without positive findings. Abdominal computed tomography (CT) identified an indistinct ileocecal structure with a "doughnut" sign, suggesting intussusception of the small intestine (Fig. 1a). However, the patient showed no typical symptoms of intussusception, such as abdominal pain and nausea. The first capsule endoscope was subsequently performed and revealed a protruding lesion with bleeding in the distal ileum (Fig. 1b). After several days of conservative treatment, hematochezia completely ceased and the patient was discharged at her request.

**Fig. 2:**
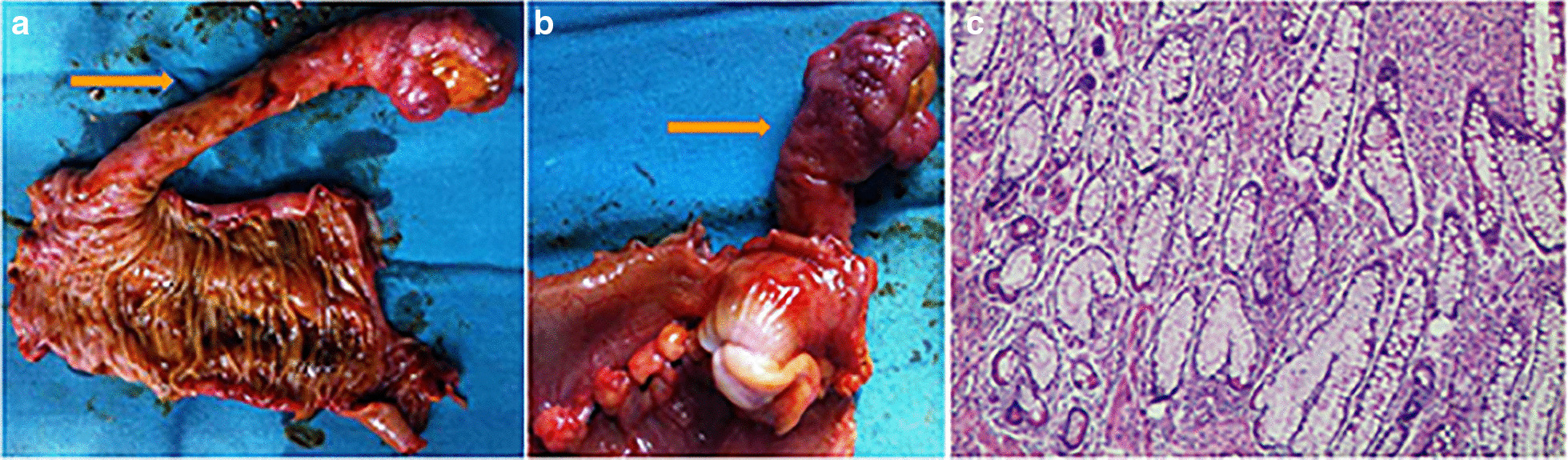
Surgical specimens (**a**, **b**) (yellow arrows) and pathological examination (**c**)

Nevertheless, she suffered a recurrence of hematochezia three days later after being discharged. The second capsule endoscope was performed and found the protruding lesion again (Fig. 1c). Following surgical consultation, the patient was referred for resection of the lesion. Intraoperatively, a 6*2 cm SIDC was discovered inside the distal ileum, 70 cm proximal to the ileocecal valve (Fig. 2a and 2b) and thus a partial resection of the small intestine was performed. Pathologic examination confirmed the diagnosis of SIDC characterized by enteric mucosa and muscular layers in the cyst wall (Fig. 2c). The patient was discharged for the second time without any complications and remained symptom-free throughout a 7-year follow-up period.

## Discussion and conclusions

EDCs are defined as cystic or tubular structures with well-developed smooth muscle walls and mucous surfaces [4]. The majority of EDCs occur before the age of two as an acute intestine obstruction, and a minority remain asymptomatic until adulthood [5]. They are found throughout the alimentary tract from the mouth to the anus. About 33% of EDCs occur in the foregut, 56% in the midgut, and 11% in the hindgut [6]. Additionally, SIDCs can be associated with all three subtypes: duodenal (2–12%), jejunal (50%), and ileal (44%) [3].

EDC has three characteristics: (a) a close attachment to the digestive tract; (b) a smooth muscle layer in its wall; and (c) a mucosal epithelial lining that resembles any part of the digestive tract [7]. Furthermore, it should share the blood supply with the native digestive tract. Most EDCs are not communicating with the native digestive tract. Our case showed a communicating and tubular cyst rarely within the inner side of the ileum in adults.

EDC patterns can be categorized into three types based on anatomy: saccular (spherical) cysts, tubular cysts, and small intramural cysts [8]. Saccular type is the most common type and not normally communicating with the native digestive tract. Tubular cysts are more commonly found in the colon. Small intramural cysts often occur near or at the ileocecal valve and protrude into the enteric lumen [8]. Coincidentally, our case was indeed a small intramural cyst.

The pathogenesis of EDC has not been fully understood, and many hypotheses have been raised, such as partial or abortive twinning, split notochord theory, embryonic diverticula, diverticular and canalization defects, intrauterine vascular accident, and environmental theory. None of these theories can adequately explain all known EDCs [9]. Partial or abortive twinning [10] could explain duplicate abnormalities of the head, upper alimentary tract, hindgut, and lower genitourinary tract. The split notochord theory [11] suggests that abnormal separation of the notochord from the gut endoderm may cause dorsal enteric duplications or diverticula [12] and this can account for the 15% of EDCs with associated vertebral defects. The aberrant luminal recanalization theory [13] speculated that enteric duplications resulted from incomplete vacuolization. Steiner et al*.* [14] hypothesized that pathological events predate torsion or some vascular accident at the proximal end of the diverticulum. Such an event could have detached it from the enteric wall, resulting in a completely isolated EDC. The intrauterine vascular accident theory [15] suggests that EDCs, like small intestine atresia, arise as the result of focal areas of vascular insufficiency secondary to fetal stress and anoxia. Mellish et al*.* [16] proposed their environmental theory in 1961, suggesting that trauma or hypoxia could induce EDCs and twinning in lower orders.

The manifestations of EDCs often vary based on the different locations in the digestive tract. Most of them are asymptomatic and some have symptoms, including feeding intolerance, abdominal pain, nausea, acute intestine obstruction, perforation, hematochezia, and palpable abdominal mass according to physical examination [6]. Among these, abdominal pain and melena are the most common symptoms [17]. The accumulation of secretions within the EDC can cause intense pain and potential obstruction due to compression of the adjacent enteric lumen. [18] The mass effect over adjacent structures may also lead to obstruction of the vena cava, biliary tree, or ureter, resulting in hydronephrosis. It is a population that is not detected in early childhood and is later diagnosed in adulthood. Subsequent diagnosis is sometimes made by chance during imaging or laparotomy [19]. Hematochezia is the protruding manifestation of our case due to the bleeding of SIDC. The age of our case is also extremely rare, as most similar cases were reported in childhood.

It is generally difficult to establish a confirmed diagnosis of EDC. X-ray results may be normal as the intestines are not being manifested very well and contrast examination is helpful to show the communication between the EDC and the native digestive tract [20]. A diagnosis of EDC can be achieved by ultrasound with typical inner echogenic mucosal and outer hypoechoic muscle layers [21]. CT and magnetic resonance imaging (MRI) have the advantage of three-dimensional imaging and are effective methods for differential diagnosis, such as Meckel’s diverticulum, appendicitis, choledochal cyst, and intussusception [22]. Although our diagnosis was mistaken as intussusception but not SIDC based on the “doughnut” sign revealed on CT imaging, our patient didn’t have acute severe abdominal pain which was the typical symptom of intussusception. We therefore performed two capsule endoscopes in order to try our best to find the causes of bleeding and revealed a protruding lesion with bleeding in the distal ileum.

As for the application of capsule endoscopy in similar cases, we have several issues to emphasize. First, the characteristic CT findings of intussusception include: (1) a thickened segment of the bowel due to the invagination of the intussusceptum into the intussuscipiens; (2) an eccentrically located lucency representing the mesentery of the intussusceptum and intraluminal air sandwiched between the two layers of the telescoped bowel; and (3) a mass, which usually represents the leading point [23]. Given that SIDCs were generally located outside the enteric cavity, differential diagnosis with intussusceptions is easy to make. However, our case was rarely located inside the enteric cavity so that it can also be showed with the “doughnut” sign on CT imaging and therefore further examinations were required. Second, we should pay attention to the risk of capsule endoscopy in patients with the “doughnut” sign of suspected intussusception on CT scans. We found that there was no report regarding the application of capsule endoscopy to intussusception. Intussusception is commonly associated with symptoms of acute severe abdominal pain and abdominal mass and it is relatively easy to be diagnosed by X-ray with no further demand for capsule endoscopy. Nevertheless, we should be aware that it is also risky to perform capsule endoscopy if the diagnosis is highly likely to be intussusception. In our case, we adopted capsule endoscopy because of the discrepancy between the patient's symptoms and the putative diagnosis of intussusception. Third, we should keep in mind the contraindications of capsule endoscopes, such as known or suspected gastrointestinal obstruction/obstacles, fistula, relevant (small bowel) diverticulosis, slow gastric emptying, and swallowing disorder (dysphagia) that may result in capsule retention [24]. A systematic review of 22,840 capsule endoscope examinations found an overall retention rate was about 1.4% and the retention rate in obscure gastrointestinal bleeding was approximately 1.2%. Of the retained capsules, 58.7% were removed surgically, 12.5% endoscopically, or passed either spontaneously or after medical treatment in 15.8% [25].

Once the diagnosis of EDC has been made, treatment is necessary to alleviate symptoms and prevent potentially serious complications, including intestine obstruction, intussusception, or hematochezia. The treatment approach for most EDCs is surgical resection. Resection can be performed through traditional open access approaches, as well as thoracoscopic and laparoscopic approaches. Important principles also include the identification and preservation of the blood supply to the native bowel [26].

Duplication cysts of the jejunum and ileum are the most common EDC types. Generally, cystic lesions are easily resected. Tubular lesions are often complex with the involvement of surrounding structures. Consequently, a portion of the native bowel must be resected along with duplication and primary anastomosis performed [27]. EDCs may be entirely excised by enucleation or resection if a noncontiguous cyst with a segregated blood supply is found. Others require resection of a small portion of the bowel. The tubular type may raise a significant challenge.

Even though malignant transformation is extremely rare in EDCs, it needs to be carefully evaluated and addressed. Malignant transformation in SIDCs is described most frequently. If transformation change is found in SIDC, the high rate of lymphatic node metastases should be considered. Curative resections have been difficult to perform and the prognosis is generally poor [28]. There are also reports about carcinomas arising in other EDCs. Liu et al*.* reported a case report of peritoneal metastatic adenocarcinoma possibly due to a gastric duplication cyst with unsuccessful resection and the ruptured cyst contaminating the peritoneal cavity. It emphasized the importance of accurate preoperative diagnosis and optimal surgical management for gastric duplication cyst due to the potential existence of malignant transformation in adult patients with gastric duplication cysts [29]. Lee et al*.* reported the first case of papillary adenocarcinoma derived from colon duplication [30].

As such, SIDC may be the most likely cause of recurrent hematochezia in our case and should be resected to prevent the recurrence of hemorrhage after surgical consultation. Exploratory resection was successfully conducted and revealed a lesion located inside the enteric cavity. Pathological examination confirmed the definitive diagnosis of SIDC.

We reported an extremely rare case of SIDC located inside the distal ileum cavity in an adult female patient with recurrent hematochezia, which should be considered in the differential diagnosis of the "doughnut" sign on radiological images.

## Data Availability

All data generated or analyzed during this study are included in this published article.

## Abbreviations

EDC: Enteric duplication cyst
SIDC: Small intestine duplication cyst
CEA: Carcinoembryonic antigen
CA19-9: Carbohydrate antigen 19–9
CT: Computed tomography
MRI: Magnetic resonance imaging
